# Treatment of COVID-19: implications for antimicrobial resistance in Africa

**DOI:** 10.11604/pamj.supp.2020.35.23713

**Published:** 2020-07-20

**Authors:** Chinwe Juliana Iwu, Portia Jordan, Ishmael Festus Jaja, Chidozie Declan Iwu, Charles Shey Wiysonge

**Affiliations:** 1 Department of Nursing and Midwifery, Faculty of Medicine and Health Sciences, Stellenbosch University, Cape Town, South Africa,; 2 Department of Livestock and Pasture Sciences, Faculty of Science and Agriculture, University of Fort Hare, Alice 5700, South Africa,; 3 School of Health Systems and Public Health, Faculty of Health Sciences, University of Pretoria, Pretoria 0001, South Africa,; 4 Cochrane South Africa, South African Medical Research Council, Cape Town, South Africa,; 5 Division of Epidemiology and Biostatistics, Stellenbosch University, Cape Town, South Africa,; 6 School of Public Health and Family Medicine, University of Cape Town, Cape Town, South Africa

**Keywords:** COVID-19 treatment, antimicrobial resistance, coronavirus, Africa

## Abstract

There is currently no approved pharmaceutical product for the treatment of COVID-19. However, antibiotics are currently being used for the management of COVID-19 patients in many settings either treat to co-infections or for the treatment of COVID-19 itself. In this commentary, we highlight that the increased rates of antimicrobial prescribing for COVID-19 patients could further worsen the burden of antimicrobial resistance (AMR). We also highlight that though AMR is a global threat, Africa tends to suffer most from the consequences. We, therefore, call on African countries not to lose sight of the possible implications of the treatment of COVID-19 on AMR and a need to redouble efforts towards the fight against AMR while dealing with the pandemic.

## Commentary

Currently, no pharmaceutical products or vaccines are safe and effective for the treatment or prevention of COVID-19. However, antibiotics are currently being used for the management of COVID-19 patients in many settings either to treat co-infections or for the treatment of COVID-19 itself. The latter is mostly used under clinical trials. A recent study has shown that the majority (72%) of individuals with COVID-19 who were hospitalized, received antibiotics, and only a fraction (8%) actually had co-infection [1]. This implies that antibiotics may not be necessary in many instances. The increased rates of antimicrobial prescribing for COVID-19 patients could further worsen the spread of antimicrobial resistance (AMR) [2]. It is essential to highlight the possibility of its impact on the development and spread of antimicrobial resistance (AMR).

**Antimicrobial resistance:** global and African perspective: it is already known that AMR is a global threat that has impacted the success of the treatment of infectious diseases and typically associated with high morbidity and mortality rates. The emergence of AMR not only makes treating life-threatening infections more complicated, but it also jeopardizes the foundation of modern health care [3]. It has been projected that by 2050, diseases caused by multidrug-resistant organisms will become the leading cause of death worldwide [3]. While the emergence and spread of AMR occur through natural means, it is facilitated mainly by excessive use and misuse of antimicrobials [3]. Africa has a high burden of infectious diseases, which points to a scenario of extensive use of antibiotics [4]. Also, the weak health systems, the poverty level in Africa, further increases the consequences of AMR. Infact, AMR has been reported among the common disease pathogens within this region, including HIV, malaria, tuberculosis, typhoid, cholera, meningitis, gonorrhea, and dysentery [4,5]. Patients in this region are often prone to life-threatening complications due to the limited access to accurate diagnosis and adequate antimicrobial treatment [6]. Antimicrobial resistance in the African region is further amplified by the indiscriminate use of antimicrobials by consumers, especially those who purchase them over the counter, without proper prescriptions [6]. Furthermore, it is important to note that between 2000 and 2015, antibiotic consumption in 76 countries of the world increased by 65%, from 21.1 to 34.8 billion Defined Daily Doses (DDDs) [7]. This increase was mostly driven by increased consumption in low and middle-income countries [7]. It is also projected that there may be a 200% increase in antibiotic consumption by 2030 if no policy changes occur[7]. With the potential increase in the use of antimicrobials for the treatment of COVID-19, these figures may increase substantially.

**Curbing AMR in Africa, in the face of COVID-19 pandemic:** several gaps existed in the fight against AMR in the African region, before the onset of COVID-19 pandemic. Firstly, evidence-based interventions for addressing antimicrobial resistance in Africa are scarce, especially those adopting the one health approach. Secondly, only a few countries have a national AMR surveillance system that generates routine representative robust data on antimicrobial use and resistance. Only a few countries, like South Africa, have AMR policies and strategic plans in place [4]. Thirdly, antimicrobial stewardship is one of the most effective measures for addressing AMR. Antimicrobial stewardship programs aid in promoting adherence to clinical treatment guidelines, help delay the emergence of AMR and improve health outcomes among patients already infected with AMR organisms [6]. A Cochrane review has shown a high certainty of evidence that interventions such as adherence to antibiotic policy, audit, and feedback among physicians improved prescribing practices and decreased the duration of antibiotic treatment [8]. However, antimicrobial stewardship is still far from being a reality in Africa. A recent systematic review showed that antimicrobial stewardship is yet to be implemented in many African countries. Also, the majority of African countries are yet to align with global efforts to combat AMR [9]. While the fight against COVID-19 in Africa continues, and with these gaps in mind, some questions may be worthy of consideration: “What impact will COVID-19 have on the burden of AMR in Africa?” Should there be a contingency plan put in place to curb the possible increase in the occurrence of AMR? These questions need to be considered for discussion by different stakeholders including, clinicians, health systems, scientists, academic institutions, pharmaceutical companies, regulatory bodies, international organizations, policy-makers, government agencies, researchers, amongst others.

**Recommendations:** the World Health Organization (WHO) has released an updated interim guidance for the clinical management of COVID-19 on 27 May 2020, taking into consideration the latest evidence on rates of co-infection [2]. The guidance discourages the use of antibiotics in all patients with mild and moderate COVID-19 unless there is clinical suspicion of a bacterial infection. Antibiotics should mostly be reserved for those suffering from severe COVID-19. Clinicians and other health professionals must consider this. Secondly, countries in Africa should hasten the process of developing national action plans to curb AMR and should incorporate management of COVID-19 into such plans. These plans should include policies that will discourage overutilization of antimicrobial agents by health professionals and consumers in Africa. There is also a need to enforce antibiotic use regulation in Africa. Such plans need to be backed up by a strong political will. Also, there is a need for the development of evidence on strategies for preventing and managing AMR in the region. The establishment of regional platforms for shared experience across African countries should also be explored [4]. Additionally, increasing the competence of health workers attending to COVID-19 patients through targeted training and the integration of antimicrobial stewardship into the pandemic response will help minimize potential emergence of AMR during the pandemic [10]. Local guidelines should continually incorporate the WHO guidance on the use of antimicrobials in managing COVID-19 and healthcare workers involved in managing COVID-19 patients should have access to these guidelines. More awareness should be created across the healthcare community, including the public and private sectors.

## Conclusion

In conclusion, while African countries make efforts towards controlling the spread of COVID-19, we should not lose sight of the possible implications of the treatment of COVID-19 on AMR. The war against AMR is yet to be won; hence, there is an urgent need for African countries to redouble the fight against AMR while dealing with the pandemic.
